# COPD is accompanied by co-ordinated transcriptional perturbation in the quadriceps affecting the mitochondria and extracellular matrix

**DOI:** 10.1038/s41598-018-29789-6

**Published:** 2018-08-15

**Authors:** Saffron A. G. Willis-Owen, Anna Thompson, Paul R. Kemp, Michael I. Polkey, William O. C. M. Cookson, Miriam F. Moffatt, Samantha A. Natanek

**Affiliations:** 1 Centre for Genomic Medicine, National Heart and Lung Institute, Imperial College London, SW3 6LY London, United Kingdom; 2 Respiratory Sciences, National Heart and Lung Institute, Imperial College London, SW3 6NP London, United Kingdom

## Abstract

Skeletal muscle dysfunction is a frequent extra-pulmonary manifestation of Chronic Obstructive Pulmonary Disease (COPD) with implications for both quality of life and survival. The underlying biology nevertheless remains poorly understood. We measured global gene transcription in the quadriceps using Affymetrix HuGene1.1ST arrays in an unselected cohort of 79 stable COPD patients in secondary care and 16 healthy age- and gender-matched controls. We detected 1,826 transcripts showing COPD-related variation. Eighteen exhibited ≥2fold changes (*SLC22A3*, *FAM184B*, *CDKN1A*, *FST*, *LINC01405*, *MUSK*, *PANX1*, *ANKRD1*, *C12orf75*, *MYH1*, *POSTN*, *FRZB*, *TNC*, *ACTC1*, *LINC00310*, *MYH3*, *MYBPH* and *AREG*). Thirty-one transcripts possessed previous reported evidence of involvement in COPD through genome-wide association, including *FAM13A*. Network analysis revealed a substructure comprising 6 modules of co-expressed genes. We identified modules with mitochondrial and extracellular matrix features, of which *IDH2*, a central component of the mitochondrial antioxidant pathway, and *ABI3BP*, a proposed switch between proliferation and differentiation, represent hubs respectively. COPD is accompanied by coordinated patterns of transcription in the quadriceps involving the mitochondria and extracellular matrix and including genes previously implicated in primary disease processes.

## Introduction

Chronic Obstructive Pulmonary Disease (COPD) is a systemic disease in which extra-pulmonary manifestations contribute significantly to the morbidity and mortality of patients. Of these, skeletal muscle dysfunction represents an attractive target for treatment. Approximately 30% of COPD patients exhibit atrophy and consequent weakness of the locomotor muscles, and reduced muscle endurance is even more prevalent, with both features detectable in the quadriceps even in mild disease^1,2^.

The impairment in quadriceps endurance observed in COPD is related to reduced muscle oxidative capacity^3,4^. Patients characteristically exhibit a low proportion of oxidative, Type I (slow-twitch) fibres in the quadriceps muscle with a reciprocal increase in fast-twitch IIa (mixed oxidative/glycolytic) and IIx (glycolytic) fibres^5^. Reduced mitochondrial numbers and oxidative enzyme activity are also reported^6^. Improvements in quadriceps oxidative capacity and strength resulting from pulmonary rehabilitation translate to improvements in exercise capacity and quality of life^7^. Conversely, reduced quadriceps mass and slow-twitch fibre proportion are markers of poor prognosis independent of lung function^8,9^.

The transcriptional machinery underlying COPD related muscle disease is not well defined. We set out to catalogue transcription in the quadriceps in an unselected cohort of 79 stable COPD patients in secondary care and 16 healthy age- and gender- matched controls. This is the largest analysis of this kind for COPD-related muscle disease. Using these data we constructed a gene co-expression network providing novel insights into the pathophysiological processes accompanying skeletal muscle dysfunction in COPD as well as facilitating the identification of novel therapeutic targets and biomarkers.

## Results

### Participant Characteristics

The cohort comprised 4 patients with GOLD I, 24 patients with GOLD II (moderate), 32 with GOLD III (severe) and 19 with very severe (GOLD IV) COPD, as well as 16 healthy age- and gender-matched controls with a normal FEV_1_/FVC ratio. Neither group had co-existing diagnoses of chronic heart failure, neuromuscular disease, type 2 diabetes or other endocrinological disorder. By design the majority of the patient cohort and controls were ex-smokers (85% and 69% respectively), with 15% of the patients but none of the controls being current smokers. The smoking exposure of the patients was significantly greater than the controls (45 vs 3 pack-years, respectively). Consistent with the published literature, and as previously reported in this cohort, patients had a reduced Type I fibre proportion in quadriceps (28.95% versus 54.44*%, P* = 7.19E-06), an increase in the proportion of Type IIa fibres (60.11% versus 40.13*%, P = *5.78E-05), and had smaller glycolytic, Type IIx fibres. There were no significant associations between FEV_1_ and either quadriceps strength or endurance in the cohort. Participant and biopsy characteristics are detailed in full in Supplementary Information Table E1.

### COPD-related differences in peripheral muscle gene expression

At a 5% false discovery rate (FDR) 1,826 transcripts achieved significance, of which 18 carried an absolute fold change ≥2 (Table 1 and Supplementary Fig. E1a). These included genes participating in a diverse range of processes comprising satellite cell regeneration (*FST*, *CDKN1A*, and *AREG*) and differentiation, including Wnt signalling (*FST*, *FRZB*, and *C12orf75*), neuromuscular transmission (*MUSK*), energy metabolism (*FST* and *C12orf75*), stress response and inflammation (*ANKRD1*, *POSTN*, *TNC* and *AREG*) and muscle structure/composition (*MYH1*, *ACTC1*, *MYH3* and *MYBPH*). Transcriptional disturbance of these 18 genes did not appear to be restricted to individuals with a low muscle mass (FFMI) (Fig. E1b).

**Table 1:** Transcripts altered in COPD quadriceps (*vastus lateralis*) relative to healthy controls.

TC	*P*-value	Adj. *P*-value	FC	Symbol	Description	Module (MM)
8123246	1.06E-11	1.71E-07	3.35	*SLC22A3*	Cation transport	Yellow (0.78)
8099551	8.74E-09	1.02E-05	2.13	*FAM184B*	—	Yellow (0.74)
8119088	9.46E-09	1.04E-05	2.53	*CDKN1A*	Cell cycle arrest; satellite cell regeneration; DNA damage response	Turquoise (−0.75)
8105302	1.33E-08	1.28E-05	2.00	*FST*	Growth factor; Wnt signalling; satellite cell regeneration	Turquoise (−0.60)
7958724	1.86E-07	5.05E-05	−2.65	*LINC01405*	—	Turquoise (0.67)
8157173	4.27E-07	7.79E-05	2.21	*MUSK*	Neuromuscular synapse formation/transmission	Turquoise (−0.75)
7943218	2.96E-06	2.97E-04	2.08	*PANX1*	Gap junctions; potentiation of skeletal muscle contraction; ATP release; mechanosensitive	Blue (0.82)
7934979	3.31E-06	3.26E-04	2.25	*ANKRD1*	Transcription factor; monocyte stress response; apoptosis; stretch-sensitive	Blue (0.73)
7958253	1.09E-05	7.16E-04	−2.66	*C12orf75*	Wnt signalling; energy metabolism	Turquoise (0.62)
8012726	1.57E-05	9.11E-04	2.21	*MYH1*	Myosin - heavy chain (type IIx fibres)	Yellow (0.66)
7971077	2.17E-05	1.14E-03	3.53	*POSTN*	Stress response; inflammation; wound healing	Blue (0.78)
8057506	3.57E-05	1.60E-03	2.62	*FRZB*	Wnt signalling; growth, proliferation & differentiation	Brown (0.54)
8163637	4.29E-05	1.80E-03	3.03	*TNC*	Stress response; inflammation; wound healing	Blue (0.79)
7987315	1.30E-04	3.75E-03	2.50	*ACTC1*	Actin	Blue (0.77)
8068363	3.58E-04	7.92E-03	−2.03	*LINC00310*	–	Blue (−0.62)
8012787	6.88E-04	1.28E-02	2.00	*MYH3*	Myosin - embryonic	Blue (0.74)
7923534	7.73E-04	1.38E-02	2.73	*MYBPH*	Myosin binding protein; actin-myosin interaction	Blue (0.81)
8095744	3.86E-03	4.26E-02	2.01	*AREG*	Growth factor; satellite cell regeneration	Green (0.55)

The Table 1 lists transcripts differentially expressed by COPD disease status in the quadriceps (*vastus lateralis*) significant at a 5% FDR and accompanied by an absolute fold change ≥2. Abbreviations: Transcript Cluster (TC), Fold Change (FC), Module Membership (MM).

Up-regulated genes highlighted common themes of muscle stress, development, growth and differentiation, as well as neuromuscular transmission. Up-regulated genes included the embryonic isoform of myosin, *MYH3*, which is only transiently expressed in adult skeletal muscle during the early phase of muscle regeneration^10^. The only protein-coding gene to show a significant reduction in COPD at the absolute fold change ≥2 threshold was *C12orf75* (also known as adipogenesis down-regulating transcript 3 [*ADG3*]) (Table 1).

The most significant differentially expressed transcript in this data set was the extra-neuronal organic cation transporter *SLC22A3*, also known as *OCT3* (t = 7.71, adj. *P* = 1.71E-07), expressed at 3.35 fold greater levels in COPD musculature relative to healthy controls. Differential expression of *SLC22A3* and direction of effect in COPD musculature are confirmed in at least one independent population and alternative platform^11^. This confirms that *SLC22A3* over-expression is a robust feature of the quadriceps transcriptome in COPD.

### Overlap with risk genes defined through genome-wide association

Thirty genes (represented by 31 TC) found here to be differentially expressed in skeletal muscle of COPD patients (adj. *P* values ranging from 6.40E-05 to 4.91E-02) carry pre-existing evidence of allelic association with COPD or intermediate traits, predominantly pulmonary, through genome-wide association^12^ (GWA, Table 2). Included amongst these genes is the widely replicated COPD susceptibility gene and mediator of lung function *FAM13A*^13–19^, 1.34 fold up-regulated in COPD musculature (adj. *P* = 6.40E-05) and the lysophosphatidic acid (LPA) receptor *LPAR1* (adj. *P = *6.91E-05, Fold Change [FC] 1.38) associated with post bronchodilator FEV_1_ in COPD^20^. These data support a role for a subset of GWA-defined candidate genes in COPD and indicate their participation in pathogenic processes that extend beyond the context of the lungs (Table 2).

**Table 2:** Differentially expressed genes previously reported in the EBI GWAS catalogue under the search term ‘chronic obstructive pulmonary disease’.

Reported gene	TC	Adj. P-Value	Module	GWAS trait	GWAS reference
*PLCE1*	7929388	1.24E-03	blue	Lifetime average cigarettes per day in chronic obstructive pulmonary disease	^43^
*LINC00310*	8068363	7.92E-03	blue	Percentage gas trapping	^44^
*SCFD2*	8100347	1.86E-02	blue	Lifetime average cigarettes per day in chronic obstructive pulmonary disease	^43^
*C5orf56*	8107934	2.85E-02	blue	Lung function (FEV1/FVC)	^19^
*KCNQ5*	8120654	2.35E-04	brown	Lung function (FEV1/FVC)	^19^
*RRM2B*	8152133	2.07E-02	brown	Chronic obstructive pulmonary disease	^16^
*PAPD4*	8106534	4.34E-02	brown	Post bronchodilator FEV1/FVC ratio in COPD	^20^
*ABCC4*	7972297	4.66E-02	brown	Airway responsiveness in chronic obstructive pulmonary disease	^45^
*LPAR1*	8163257	6.91E-05	green	Post bronchodilator FEV1 in COPD	^20^
*ABI3BP*	8089145	4.51E-03	green	Post bronchodilator FEV1 in COPD	^20^
*DLC1*	8149413	8.86E-03	grey	Emphysema imaging phenotypes; Percentage gas trapping	^44^
*RARB*	8078286	4.91E-02	grey	Airway responsiveness in chronic obstructive pulmonary disease; Chronic obstructive pulmonary disease	^16, 45^
*PDZD2*	8104693	3.41E-03	red	Chronic obstructive pulmonary disease	^46^
*FAM13A*	8101728	6.40E-05	turquoise	Chronic obstructive pulmonary disease; Chronic bronchitis and chronic obstructive pulmonary disease; Chronic obstructive pulmonary disease (moderate to severe); Chronic obstructive pulmonary disease (severe); Lung function (FEV1/FVC)	^13– 19^
*CACNA2D3*	8080578	2.38E-04	turquoise	Lung function (FEV1/FVC)	^19^
*MN1*	8075126	1.23E-03	turquoise	Lung function (FEV1)	^19^
*CDH23*	7928218	1.55E-03	turquoise	Age at smoking initiation in chronic obstructive pulmonary disease	^43^
*NNT*	8105153	2.54E-03	turquoise	Post bronchodilator FEV1/FVC ratio in COPD	^20^
*THSD4*	7984588	3.67E-03	turquoise	Chronic obstructive pulmonary disease; Lung function (FEV1/FVC)	^16, 19^
*KCNS3*	8040458	5.69E-03	turquoise	Lung function (FEV1); Lung function (FEV1/FVC)	^19^
*MICAL3*	8074274	1.20E-02	turquoise	Lung function (FEV1)	^19^
*MLYCD*	7997525	1.36E-02	turquoise	Response to bronchodilator in chronic obstructive pulmonary disease (change in FEV1)	^47^
*PLXNA4*	8142997	1.63E-02	turquoise	Response to bronchodilator in chronic obstructive pulmonary disease (change in FEV1)	^47^
*AZIN1*	8152222	1.66E-02	turquoise	Airway imaging phenotypes	^44^
*VWA8*	7971246	2.41E-02	turquoise	Local histogram emphysema pattern	^48^
*ARMC2*	8121370	2.91E-02	turquoise	Chronic obstructive pulmonary disease; Lung function (FEV1/FVC)	^16, 19^
*MICAL3*	8074286	3.31E-02	turquoise	Lung function (FEV1)	^19^
*CDH13*	7997504	3.46E-02	turquoise	Response to bronchodilator in chronic obstructive pulmonary disease (change in FEV1)	^47^
*AGFG1*	8048847	3.88E-02	turquoise	Airway hyperresponsiveness	^49^
*ABLIM2*	8099279	2.70E-02	yellow	Post bronchodilator FEV1 in COPD	^20^
*PPM1L*	8083749	4.65E-02	yellow	Post bronchodilator FEV1/FVC ratio in COPD	^20^

The table lists transcripts differentially expressed by COPD disease status in the quadriceps (*vastus lateralis*) at a 5% FDR and catalogued under the annotation ‘chronic obstructive pulmonary disease’ in the NHGRI-EBI GWAS database^12^ as accessed on 12/06/2018.

Abbreviations: Transcript Cluster (TC), Fold Change (FC), Genome-Wide Association Study (GWAS), Module Membership (MM).

### Peripheral muscle gene co-expression patterns

Patterns of co-ordination amongst differentially expressed genes were sought through network analysis (Weighted Gene Co-expression Network Analysis, WGCNA)^21^ yielding 6 modules ranging in size from 39 to 899 members (Table 3). A total of 195 transcripts remained unassigned suggesting they act in a less co-ordinated manner. Modules were placed within a functional, cellular and clinical context through patterns of enrichment for Gene Ontology (GO) terms and publicly catalogued DisGeNET disease-gene associations (DGA). Modules summaries are given in Table 3.

**Table 3:** COPD-related peripheral muscle co-expression modules.

Module	N	Peak MM	Top GO BP (adj. P value)	Top GO CC (adj. P value)	Top DGA (adj. P value)
Blue	264	TC 8051443 (0.88), *STRN*	NS	NS	umls:C0349782 Ischemic cardiomyopathy (3.29E-04)
Brown	231	TC 8044161 (0.82),–	GO:0048193 Golgi vesicle transport; GO:0051603 Proteolysis involved in cellular protein catabolic process (1.76E-02)	GO:0044446 Intracellular organelle part; GO:0044422 Organelle part (4.80E-04)	NS
Green	93	TC 8089145 (0.87), *ABI3BP*	GO:0030199 Collagen fibril organization (7.89E-04)	GO:0005615 Extracellular space (7.60E-08)	umls:C0034069 Pulmonary Fibrosis (1.82E-05)
Red	39	TC 7919349 (0.86),–	GO:0006614 SRP-dependent co-translational protein targeting to membrane; GO:0006613 Co-translational protein targeting to membrane; GO:0045047 Protein targeting to ER; GO:0072599 Establishment of protein localization to endoplasmic reticulum (1.97E-04)	GO:0022626 Cytosolic ribosome (1.83E-03)	NS
Turquoise	899	TC 7991374 (0.91), *IDH2*	GO:0055114 Oxidation-reduction process (3.04E-43)	GO:0044429 Mitochondrial part (5.35E-53)	umls:C0023264 Leigh Disease (1.01E-08)
Yellow	105	TC 8046646 (0.84), *OSBPL6*	NS	GO:0043292 Contractile fiber (1.92E-10)	NS
Unassigned	195				
Sum	1826				

The table summarises detected gene co-expression modules. The number of module members is shown as N, the transcript showing the highest module membership is reported with module membership (MM) shown in brackets. Functional, cellular and clinical context is provided through top enriched gene and disease ontology terms, with the false discovery rate controlled at 5%. Tied terms are reported. Abbreviations: Module Membership (MM), Gene Ontology (GO), Biological Process (BP), Cellular Component (CC), Transcript Cluster (TC), Not Significant (NS), Disease-Gene Association (DGA), Unified Medical Language System (UMLS), Disease Ontology ID (DOID).

The largest group of co-expressed genes (899 members, 49.23% of differentially expressed genes) was the turquoise module, which showed pronounced enrichment for localisation in constituent parts of the mitochondrion (GO:0044429 adj. *P* = 5.35E-53) and related biological processes (GO:0055114 oxidation-reduction process, adj. *P* = 3.04E-43; GO:0045333 cellular respiration, adj. *P* = 3.68E-41). Likewise, DGA enrichment peaked at Leigh Disease - a neurodegenerative disorder linked to defects in mitochondrial energy production (21 genes, adj. *P* = 1.01E-08, umls:C0023264). Cardiomyopathies were also highly over-represented (adj. *P* = 7.99E-06, umls:C0878544) with 46 disease-associated genes present in the turquoise module. A full listing of enriched DGA is given in Table E3.

The green module demonstrated a pattern of enrichment for GO terms indicative of localisation in extracellular space (adj. *P* = 7.60E-08) and participation in processes relating to the extracellular matrix (ECM): collagen fibril organisation (GO:0030199, adj. *P* = 7.89E-04) and extracellular matrix organisation (GO:0030198, adj. *P* = 2.39E-03). Eight genes in the green module possessed curated associations to osteoporosis (adj. *P* = 4.90E-02), a frequent co-morbidity of COPD.

### Gene co-expression modules differentially associate with COPD disease features

**Figure 1:**
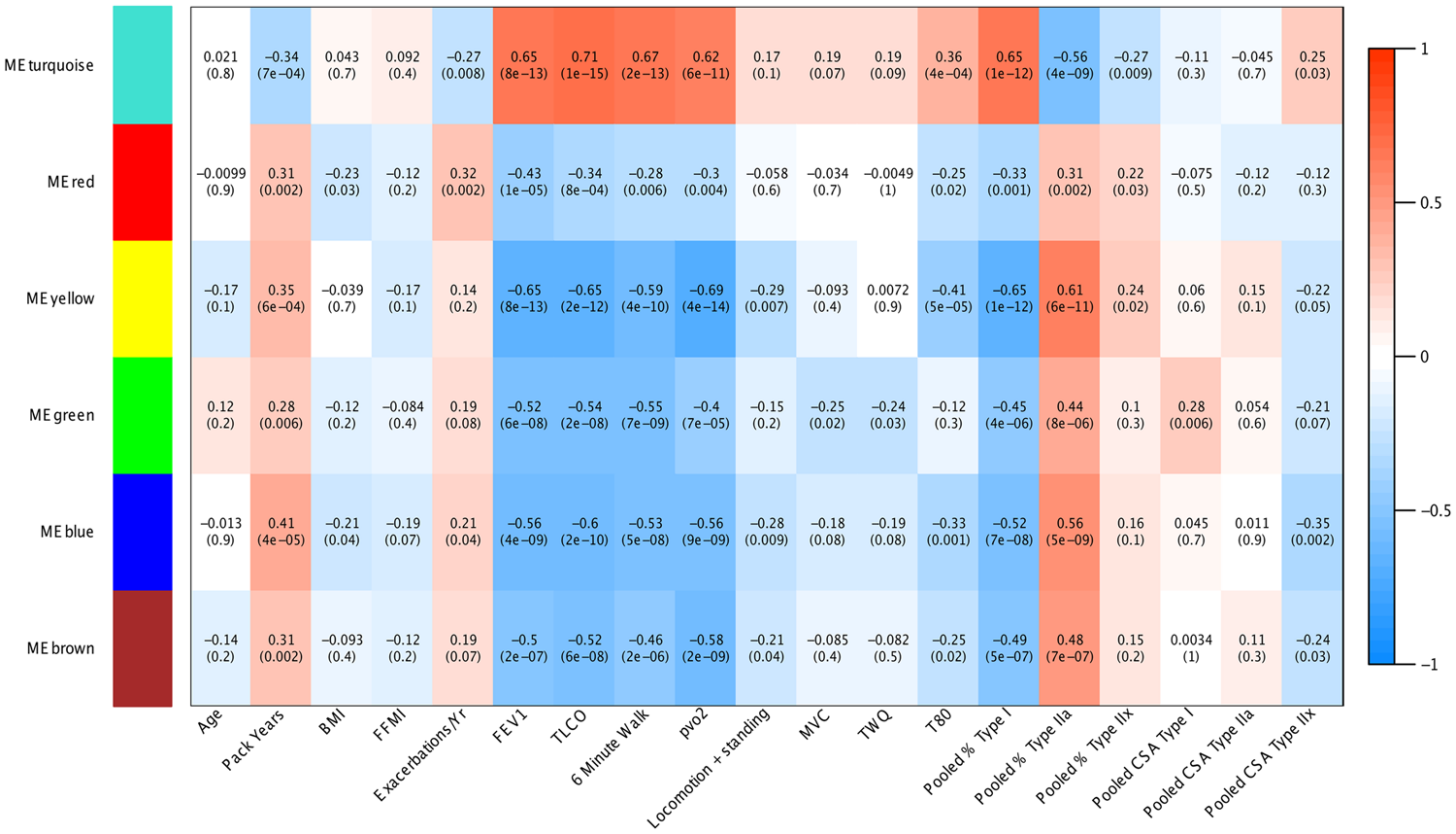
Correlations between module eigengenes and COPD-related quantitative clinical traits. Cells are colour-coded as per the colour legend, indicating the direction and magnitude of the correlation. Cells detail the correlation statistic and the associated raw unadjusted *P*-value. Traits are shown in columns, Module Eigengenes (ME) are shown in rows. Module Eigengenes are defined as the first principal component of each module, and are considered representative of the overall module expression profile.

We quantified the relationship between gene co-expression modules and clinical features through correlation (Fig. 1). The turquoise (mitochondrial) and yellow modules exhibited largely opposing patterns of effect, yielding the strongest relationships with pulmonary function (FEV_1_ and TL_CO_), exercise performance (6 minute walk and peak VO_2_ on incremental cycle ergometry), quadriceps endurance (T_80_) and fibre type ratios (pooled percentage Type I, IIa and IIx) (Fig. 1). The yellow and blue modules were both negatively associated with measures of daily physical activity. The green (ECM) module showed the closest relationship with indexes of strength (maximal voluntary contraction, MVC and involuntary T_W_Q), though only the relationship with MVC achieved significance following a BH *P*-value adjustment (adj. *P* = 3.49E-02). Of all modules, the red module showed the closest association with the frequency of exacerbations (adj. *P* = 5.33E-03) while the blue module carried the strongest association with tobacco smoke exposure (pack years, adj. *P* = 1.49E-04). Following *P*-value adjustment, no module demonstrated a significant relationship with age, BMI or FFMI. In terms of muscle wasting, the green (ECM) module was positively correlated with Type I fibre cross-sectional area (CSA, adj. *P* = 1.49E-02) whilst the blue module was negatively related to Type IIx CSA (adj. *P* = 5.39E-03) (Fig. 1).

### Using module membership to prioritise genes

Within modules, transcripts were sorted by their degree of module membership (MM), describing the strength and direction of the relationship between a transcript and module. This allowed the prediction of hubs and prioritisation of genes for further investigation.

Membership of the green (ECM) module peaked for the gene *ABI3BP* that encodes the extracellular matrix ABI3 binding protein formally known as TARSH. Within the turquoise (mitochondrial) module, the highest MM was observed for *IDH2* that encodes a mitochondrial isocitrate dehydrogenase. Also within this module is the *SIRT3* gene. A regulatory relationship has been previously reported between IDH2 and SIRT3. Here we observed down-regulation of both *IDH2* (adj. *P* = 1.06E-06, FC −1.33) and *SIRT3* (adj. *P* = 1.73E-04, FC −1.28) in the COPD peripheral musculature.

**Figure 2:**
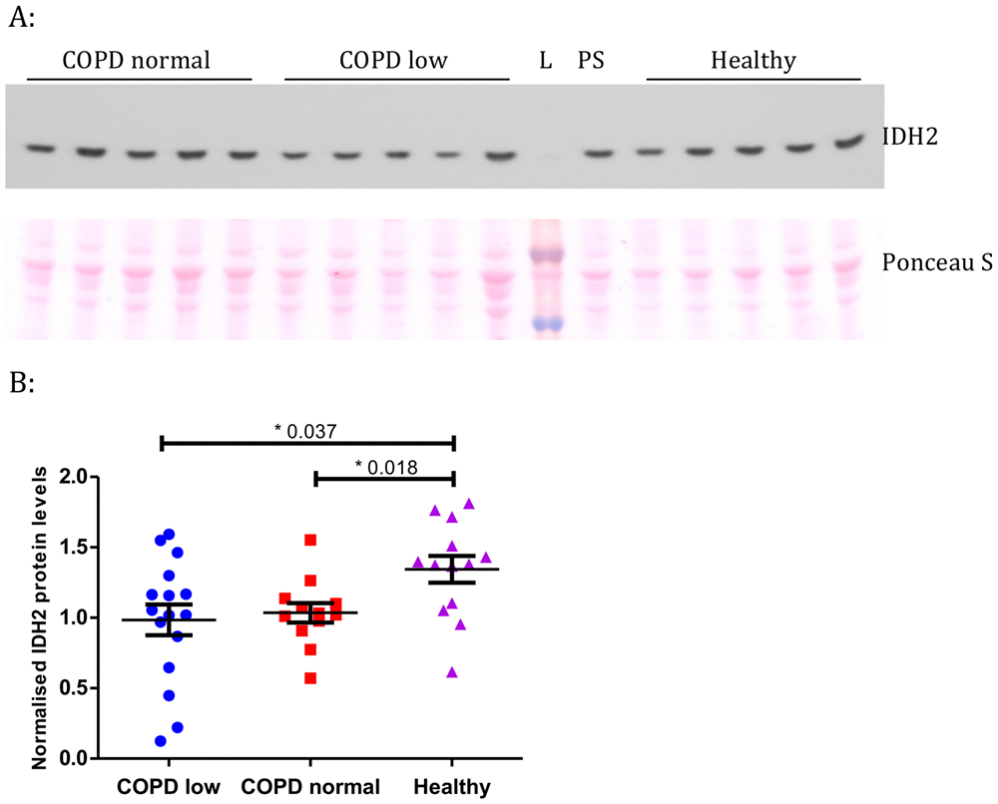
Western blot data for IDH2 using quadriceps muscle specimens from COPD patients and healthy controls. (**A**) Representative IDH2 Western blot image. See Supplementary Fig. E2 for the full-length blot. Ponceau S was used as a loading control, see Supplementary Fig. E3 for full-length image. L = ladder, PS = protein standard. (**B**) IDH2 protein levels measured by Western blotting in COPD patients (N = 30) and healthy controls (N = 13). COPD low: patients with a low (<27%) slow-twitch fibre proportion according to normal ranges, COPD normal: patients with a slow-twitch fibre proportion within the normal range^5^.

Single fibre studies indicate that *IDH2* may represent part of the mitochondrial specialisation of Type I muscle fibres and as such reduced abundance in COPD may reflect variation in fibre type proportions. Given that the turquoise (mitochondrial) module represents the single largest module detected we explored this hypothesis using R^2^ decomposition, estimating the relative importance of fibre type and disease state in predicting *IDH2* abundance. A model including disease state, Type I, Type IIa and Type IIx fibre proportions explained 45.37% of variance in *IDH2* expression. Of this, 43.09% was attributed to disease state (95% CI, 100 bootstrap replicates: 24.30–62.94%), whilst 33.8% was attributed to Type I fibre proportions (16.27–50.60%), 17.8% to Type IIa (5.02–31.90%) and 5.32% to Type IIx (0.09–17.84%). Next we measured IDH2 protein levels in a subset of the cohort for whom quadriceps biopsy total protein extracts were available (for the accompanying demographics see supplementary Table E2). IDH2 protein levels were significantly lower in COPD cases relative to healthy controls (*P* = 0.01), but not significantly different between COPD patients with low Type I fibre proportions and COPD patients with normal^5^ fibre type proportions (*P* = 0.95, Fig. 2). These data suggest that the influence of disease state on *IDH2* abundance is not restricted to an effect on fibre type proportions but that additional mechanisms influence *IDH2* abundance.

## Discussion

In the current study we set out to catalogue transcriptional perturbations that accompany COPD in the quadriceps, and place changes observed within a systems-level context. We have identified 1,826 transcripts the abundance of which varies by disease state. Eighteen show large magnitude fold changes and 31 carry pre-existing evidence of allelic association via GWA with COPD, including pulmonary manifestations. We have identified 6 modules of tightly co-expressed genes that differentially associate with parameters of disease and show contrasting patterns of enrichment for cellular localisation and disease-gene associations. Membership of these modules reveals key hub genes providing a metric for the future prioritization of targets. These data provide a snapshot of the transcriptional phenomena that accompany COPD in the quadriceps but cannot differentiate causal from reactive or compensatory mechanisms, and require future assessment of preservation in independent populations and across GOLD stages.

The genes demonstrating the most pronounced disease-associated changes in abundance highlight themes of inflammation and regeneration, neuromuscular junction (NMJ) (in)stability and metabolic stress. The most significant differentially expressed transcript in this data set was the extra-neuronal organic cation transporter *SLC22A3*, exhibiting a >3-fold difference in abundance between healthy controls and COPD patients. *SLC22A3*, also known as *OCT3*, participates in the disposition and clearance of xenobiotics and endogenous organic cations such as catecholamines. *SLC22A3* demonstrates a particular preference for histamine^22^, an established regulator of micro-circulation and key mediator of post-exercise skeletal muscle recovery and exercise-induced fatigue^23,24^. *SLC22A3* is under demonstrable genetic and epigenetic control and is sensitive to pharmacological inhibition. Functional allelic variants of *SLC22A3* affect uptake efficiency and substrate specificity and as such may be worthy of exploration in the context of COPD. In terms of skeletal muscle injury and regeneration, we document heightened expression of embryonic myosin (*MYH3*) indicating the presence of active regenerative processes. Also increased were the potent cyclin-dependent kinase inhibitor and marker of cell cycle arrest *CDKN1A* (also known as p21); the growth factor amphiregulin (*AREG*) that promotes muscle repair; and pannexin 1 (*PANX1*) a mechano-sensitive ATP channel the expression of which increases under conditions of inflammation and regeneration^25^.

In terms of NMJ (in)stability, we observe 2.21-fold up-regulation of the NMJ gene *MUSK* in COPD. This up-regulation is consistent with inactivity and functional denervation in COPD patients. With regard to metabolic stress, we observed a 2-fold elevation of *FST* (encoding follistatin) in the quadriceps of COPD patients. Circulating follistatin levels increase under conditions of energy deprivation in humans (such as exercise and prolonged fasting) and are regulated by the glucagon-to-insulin ratio^26^. In skeletal muscle follistatin induces hypertrophy and its expression in myoblasts, controlled through the Wnt/β-catenin signalling pathway, is associated with induction of myogenic differentiation^27^. In COPD, up-regulation of follistatin may represent a compensatory mechanism aiming to sustain muscle mass in response to energy deficit. The only protein-coding gene to show a large magnitude reduction in abundance was *C12orf75*, which binds the insulin receptor substrate 4 protein (IRS4) to promote insulin signalling in rat hypothalamic and human embryonic kidney cells^28^, and undergoes alternative splicing with products differentially regulating Wnt signalling^29^. This may similarly be a mechanism co-ordinating energy status and muscle growth/regeneration in COPD.

Network analysis revealed clear patterns of transcriptional co-ordination in the quadriceps. The single largest module (the turquoise module), containing over 49% of all genes differentially expressed between COPD patients and healthy controls, showed manifest enrichment for gene ontology terms relating to the mitochondria as well as strong association with measures of lung function and exercise performance. The highest-ranking hub gene within this module, *IDH2*, encodes a mitochondrial enzyme that converts isocitrate to 2-ketoglutarate, yielding NADPH. The latter is necessary for regeneration of the antioxidant glutathione and therefore key to preserving the mitochondrial redox balance and safeguard against cellular oxidative damage. Consistent with this role, down-regulation of *IDH2* has previously been shown to mediate the negative effects of hypercapnia on cellular proliferation and mitochondrial function in fibroblasts and alveolar epithelial cells^30^ as well as the negative effects of hypoxia on proliferation in glioma cells^31^. Furthermore, genetic variants in the *IDH2* locus also moderate the impact of environmental oxidant challenge on lung function^32^. Here we observe a relative reduction of *IDH2* in the peripheral musculature of COPD patients, suggesting that its cognate co-expression module may reflect a cellular reaction to stress conditions (operating through *IDH2* reduction) resulting in mitochondrial dysfunction and a reduced metabolic activity.

In contrast with the above, a second gene co-expression module (green module) with predominantly extracellular matrix features represented the only module to display a significant relationship with indices of strength (MVC) or Type I fibre CSA, indicating potential roles in atrophy and force transduction. The ECM is a dynamic, bioactive milieu providing physical and biochemical support to all tissues. Dysregulation of the ECM can yield pathological consequences including bone malformations, cancer and fibrosis^33^. In muscle the ECM has a critical role in maintaining the satellite cell niche and therefore muscle regeneration^34^. Within the green module the gene *ABI3BP* was identified as the primary hub. Previously suggested to play a role in the aetiology of COPD through GWA^20^, *ABI3BP* encodes an ECM protein postulated as a switch between proliferation and differentiation^35^ and a trigger for cellular senescence^36^. The expression of *ABI3BP* within the context of the lung was recently shown to be regulated by cis-acting genetic variants, which may be worthy of exploration in COPD due to their potential to provide novel proxies/biomarkers for ECM module function. Also present in the ECM module, and showing high levels of module membership (a concept closely related to connectivity) was *LPAR1* a gene previously connected with pulmonary features of COPD via GWA and the target of an antagonist (BMS-986020, formerly BMS-986202, AM152) currently in Phase II clinical trials for the treatment of IPF (NCT01766817). *In vitro* and mouse studies additionally suggest potential application of LPAR1 antagonists in osteoporosis, which is notable given that osteoporosis is a frequent co-morbidity of COPD with an overall mean prevalence of 35.1%^37^. *LPAR1* may therefore provide a potential means of targeting this gene co-expression module therapeutically.

There are a number of inter-related factors associated with a diagnosis of COPD that are difficult to disentangle in clinical populations, including but not limited to smoking history, systemic inflammation and physical (in)activity. We attempted to control for the acute effects of muscle activity on gene expression and any differences in activity in patients and controls immediately prior to the muscle biopsy by resting all patients for 20 minutes before the procedure. Our aim here was to define gene expression patterns that accompany COPD in the quadriceps, irrespective of the causation, given that potential therapeutic targets may fall into pathways affected by COPD itself, but also those disrupted by its associated factors. This data set does not therefore allow us allow us to distinguish causative from responsive elements of the COPD transcriptome, and further longitudinal *in vivo* and/or controlled cell line studies will be needed to define the temporal sequence of events and relative contribution of uncontrolled factors such as smoke exposure. We also acknowledge that there are a number of other non-muscle cell types, such as fibroblasts and inflammatory cells, in a muscle biopsy that will contribute to the gene expression results we present.

Our results are consistent with a recently published network analysis of transcriptomic changes in a small sample of 15 COPD patients and 12 healthy controls before and after an exercise program^38^. Key networks differentially expressed in patients and controls in that paper pre-training included those relating to mitochondrial bioenergetics and stress response/inflammation which recapitulates our findings, with COPD patients having an exaggerated remodelling response to exercise consistent with the ECM gene module we identify.

In conclusion, we provide a fine-scale catalogue of the transcriptional variation that accompanies COPD in the peripheral musculature, as well as its coarse-scale patterns of organisation and key hub genes. We focus on two modules with mitochondrial and extracellular matrix features respectively. We additionally confirm, at the level of mRNA level in the quadriceps, a variety of candidate genes previously implicated in the aetiology of COPD and its pulmonary features though allelic association thereby suggesting that a proportion of these pathogenic mechanisms may have relevance in the peripheral musculature.

## Methods

Please see Supplementary Information for further details.

### Participants, clinical phenotyping, quadriceps biopsy collection and analysis

The study was approved by the Royal Brompton & Harefield NHS Trust and Ealing and West London Mental Health Trust Research Ethics Committees (Studies 06/Q0404/35 and 06/Q0410/54). Subjects gave written informed consent. All methods were performed in accordance with the relevant guidelines and regulations. Quadriceps muscle specimens, taken by percutaneous needle biopsy, after participants had rested supine for 20 minutes, and snap frozen, were available from a convenience sample of 101 GOLD Stage I-IV COPD patients recruited from clinics at the Royal Brompton Hospital and 21 healthy controls recruited by advertisement^39^.

Physiological data pertaining to lung function, body composition (fat-free mass [FFM] and FFM index [FFMI]), quadriceps strength and endurance, exercise capacity (6-minute walk distance and peak oxygen consumption on incremental cycle ergometry protocol), physical activity (measured with an accelerometer), as well as muscle fibre proportions and fibre cross-sectional area quantified from immunostaining muscle sections were available for this cohort (described in^39^, see supplement). Low and normal FFMI was defined using the Dutch criteria (low if less than 15 kg/m^2^ for women and less than 16 kg/m^2^ in men).

### Gene expression profiling

Total RNA was extracted from frozen quadriceps specimens stored at −80 °C using the RNeasy fibrous tissue kit (Qiagen). RNA quality was determined by Bioanalyzer (Agilent), and 5 samples with a RIN (RNA Integrity Number) <6 were excluded. RNA was prepared for microarray analysis (98 COPD and 19 healthy controls) using the WT expression kit (Ambion) and GeneChip WT terminal labelling and hybridisation kit (Affymetrix). Samples were processed using the GeneTitan system and HuGene 1.1 ST 16- or 24-PEG array plates (Affymetrix).

### Statistical analysis

Sample characteristics were analysed in R. Group differences were assessed with the Welch two sample t-test or Mann Whitney U-test depending on the data distribution. Group differences in categorical data were tested with Fisher’s exact test.

Gene expression data were RMA-treated using Affymetrix Power Tools (APT, 1.16.1) and imported into R (version 3.1.2) for analysis. Pre-processing and filtering yielded 95 samples (79 COPD, 16 healthy controls) and 18,625 TC for analysis. Differential expression between healthy and COPD samples was estimated using the R package Limma (3.22.7) with a Benjamini and Hochberg^40^ *P*-value adjustment. Adjusted *P*-values are frequently referred to as *q*-values and allow control of the FDR below a given threshold. Please see the online Limma documentation for further details. Patterns of transcriptional co-ordination were sought amongst differentially expressed genes using WGCNA (1.46)^21^. The relationship between modules and quantitative clinical traits was assessed through robust biweight midcorrelation. Enrichment analyses were performed using the R packages topGO (2.20.0) and DOSE (3.0.4)^41^. To estimate the relative importance of fibre type and disease state in predicting transcript abundance, we applied the R^2^ decomposition based on the methods of Zuber and Strimmer^42^. Further details are provided in the Supplement.

### Data availability

Microarray data for samples used in this analysis have been deposited in the NCBI Gene Expression Omnibus (GEO) repository with Accession Number GSE100281.

### Western blotting and ELISA

Immunoblotting for isocitrate dehydrogenase 2 (IDH2) was performed in quadriceps muscle protein extracts from 30 COPD patients and 13 controls.

## Electronic supplementary material

Supplementary information
