# Supplementary material for: COPD is accompanied by co-ordinated transcriptional perturbation in the quadriceps affecting the mitochondria and extracellular matrix
Source: Sci Rep. 2018 Aug 15;8:12165. doi: 10.1038/s41598-018-29789-6 (PMC6093887; doi:10.1038/s41598-018-29789-6)
Supplement: Supplementary file 1 — Supplementary information [file 41598_2018_29789_MOESM1_ESM.pdf]

SUPPLEMENTARY INFORMATION

TITLE COPD is accompanied by co-ordinated transcriptional perturbation in the quadriceps affecting the mitochondria and extracellular matrix.

AUTHORS
Saffron AG Willis-Owen *#[1], Anna Thompson#[1], Paul Kemp [2], Michael I Polkey [2], William OCM Cookson [1], Miriam F Moffatt [1]†, Samantha A Natanek *[2]†.

* Corresponding authors
# Joint First Authors
† Joint Senior Authors

SUPPLEMENTARY METHODS

Participants

Patients were recruited from clinics at the Royal Brompton Hospital whilst healthy controls were recruited by advertisement.

Physiological methods

Post-bronchodilator spirometry, lung volumes by plethysmography and carbon monoxide diffusion capacity were measured according to American Thoracic Society (ATS) guidelines and arterialized capillary earlobe blood gas tensions measured. Fat-free mass (FFM) was measured in participants, after a twenty-minute period resting supine, using the bioelectrical impedance technique (Bodystat 1500, UK). The FFM index (FFMI) was calculated using a disease-specific regression equation. Quadriceps strength was assessed by supine isometric Maximal Voluntary Contraction (MVC) as described by Edwards *et al* [1]. Post-bronchodilator exercise performance was assessed with a single 6-minute walk (6MW) performed according to ATS guidelines [2] and symptom-limited incremental cycle ergometry with metabolic testing as described previously [3].

Quadriceps sampling

Percutaneous biopsy of the *vastus lateralis* (quadriceps) was performed using the Bergstrom technique [4]. Samples for RNA and protein analysis were snap frozen in liquid nitrogen whilst samples for histology were frozen in melting isopentane prior to storage at –80°C.

Measurement of quadriceps fibre CSA and fibre proportions

Frozen 10µm transverse muscle cross-sections were incubated with primary antibodies against Type I and IIa myosin and laminin followed by fluorescent-labelled secondary antibodies. Epi-fluorescence (Nikon Eclipse 800, Nikon Instruments Europe BV, Netherlands) classified fibres as Type I, IIa (IIa and IIa/IIx), IIx and hybrid I/IIa and fibre CSA was determined using Lucia 4.81 software (Laboratory Imaging, Czech Republic) for each subject. Therefore, a subject's Type I and Type II fibre proportions and median Type I and Type II fibre CSAs could be calculated. At least 101 fibres were analysed per subject [5].

Western blotting and ELISA

Total protein homogenates from quadriceps specimens were prepared by homogenising muscle samples under liquid nitrogen and re-suspending the homogenate in lysis buffer (NaCl [300mM], Tris-Base [20mM], EDTA [10mM] 2% Igepal NP40 pH 7.3) supplemented with protease and phosphatase inhibitor cocktails (Sigma, UK). Protein concentrations were measured by Bradford assay and samples were mixed with sample buffer (loading buffer and betamercaptoethanol) at a 1:1 volume ratio and boiled for five minutes at 100°C. Total protein was analysed by gel electrophoresis using NuPAGE Novex 4-12% Bis-Tris Gel (Invitrogen) and the iBlot dry transfer technique to nitrocellulose membrane (Invitrogen). Membranes were blocked in 5% non-fat dry milk for 1 hour and then incubated in the relevant primary antibody at 4°C overnight. Following an hour long room temperature incubation in the relevant secondary antibody, proteins were visualised using ECLplus (GE Healthcare) and optical densities were determined using ImageJ (http://rsbweb.nih.gov/ij/index.html). Antibodies used were: rabbit anti-IDH2 (HPA007831, Sigma) 1/200 dilution, rabbit anti-AK1 (H-90) (sc-28785, Santa Cruz Biotechnology) 1/500 dilution, and horseradish peroxidise (HRP) conjugated pig anti-rabbit IgG antibody (P0399, Dako) 1/3000 dilution. Ponceau S was used as a loading control. Between blot comparisons were enabled by loading an equal amount of protein standard on each gel.

Analysis of gene expression data

Raw data quality was assessed using arrayQualityMetrics (3.22.1). Technical batch effects were identified via Principal Variance Component Analysis (PVCA 1.6.0) and the data adjusted accordingly using ComBat (SVA Version 3.12.0) [6]. Transcript clusters (TC) forming part of the main array design were retained (n = 28,869) for analysis. Universally low expressed TC were removed. Together these criteria yielded a total of 18,625 transcript clusters for analysis. Remaining outlying samples (n = 1) were specified through hierarchical clustering and also removed resulting in 95 samples (79 COPD, 16 Healthy controls) for analysis. Differential expression between healthy and COPD samples was estimated using Limma (Version 3.22.7). P-values were adjusted for multiple testing using the Benjamini & Hochberg method [7] to control the False Discovery Rate (FDR) below 5%. Occult patterns of transcriptional co-ordination were sought amongst differentially expressed genes through Weighted Gene Co-expression

Network Analysis (WGCNA Version1.46) [8], employing a soft-threshold power of 6 and minimum module size of 30. The relationship between modules and measured traits was assessed through robust biweight midcorrelation, with the exact number of observations taken into account in *P*-value calculation. Enrichment analysis was performed on unique entrez IDs using the Bioconductor packages topGO (Version 2.20.0) for Gene Ontology and DOSE (Version 3.0.4) for Disease-Gene Associations [9]. Annotations were made using packages annotate (Version 1.46.1) and hugene11sttranscriptcluster.db (Version 8.3.1). In order to estimate the relative importance of fibre type and disease state in predicting transcript abundance, we applied the R^2 decomposition based on the methods of Zuber and Strimmer [10]. Microarray data for samples used in this analysis have been deposited in the NCBI Gene Expression Omnibus (GEO) repository with Accession Number GSE100281.

References

1 Edwards, R. H., Young, A., Hosking, G. P. & Jones, D. A. Human skeletal muscle function: description of tests and normal values. *Clin. Sci. Mol. Med.* **52**, 283-290 (1977).

2 ATS statement: guidelines for the six-minute walk test. *Am. J. Respir. Crit. Care Med.* **166**, 111-117, doi:10.1164/ajrccm.166.1.at1102 (2002).

3 Hopkinson, N. S. *et al.* Acute effect of oral steroids on muscle function in chronic obstructive pulmonary disease. *The European respiratory journal* **24**, 137-142 (2004).

4 Bergstrom, J. Percutaneous needle biopsy of skeletal muscle in physiological and clinical research. *Scand. J. Clin. Lab. Invest.* **35**, 609-616 (1975).

5 Natanek, S. A. *et al.* Heterogeneity of quadriceps muscle phenotype in chronic obstructive pulmonary disease (Copd); implications for stratified medicine? *Muscle a d Nerve* **48**, 488-497, doi:10.1002/mus.23784 (2013).

6 Johnson, W. E., Li, C. & Rabinovic, A. Adjusting batch effects in microarray expression data using empirical Bayes methods. *Biostatistics* **8**, 118-127, doi:10.1093/biostatistics/kxj037 (2007).

7 Benjamini, Y. & Hochberg, Y. Controlling the false discovery rate: a practical and powerful approach to multiple testing. *Journal of the Royal Statistical Society Series B*, 289-300 (1995).

8 Langfelder, P. & Horvath, S. WGCNA: an R package for weighted correlation network analysis. *BMC Bioinformatics* **9**, 559 (2008).

9 Yu, G., Wang, L.-G., Yan, G.-R. & He, Q.-Y. DOSE: an R/Bioconductor package for disease ontology semantic and enrichment analysis. *Bioinformatics (Oxford, England)* **31**, 608-609 (2015).

10 Zuber, V. & Strimmer, K. High-Dimensional Regression and Variable Selection Using CAR Scores. *Stat Appl Genet Mol Biol* **10**, doi:Artn 34
10.2202/1544-6115.1730 (2011).

11 Miller MR, C. R., Hankinson J, et al. . 2005;26(1):153–61. General considerations for lung function testing. *Eur Respir J.*

SUPPLEMENTARY FIGURES AND TABLES

Figure E1a: Transcript clusters differentially expressed at a 5% false discovery threshold accompanied by a minimum absolute fold change of 2.

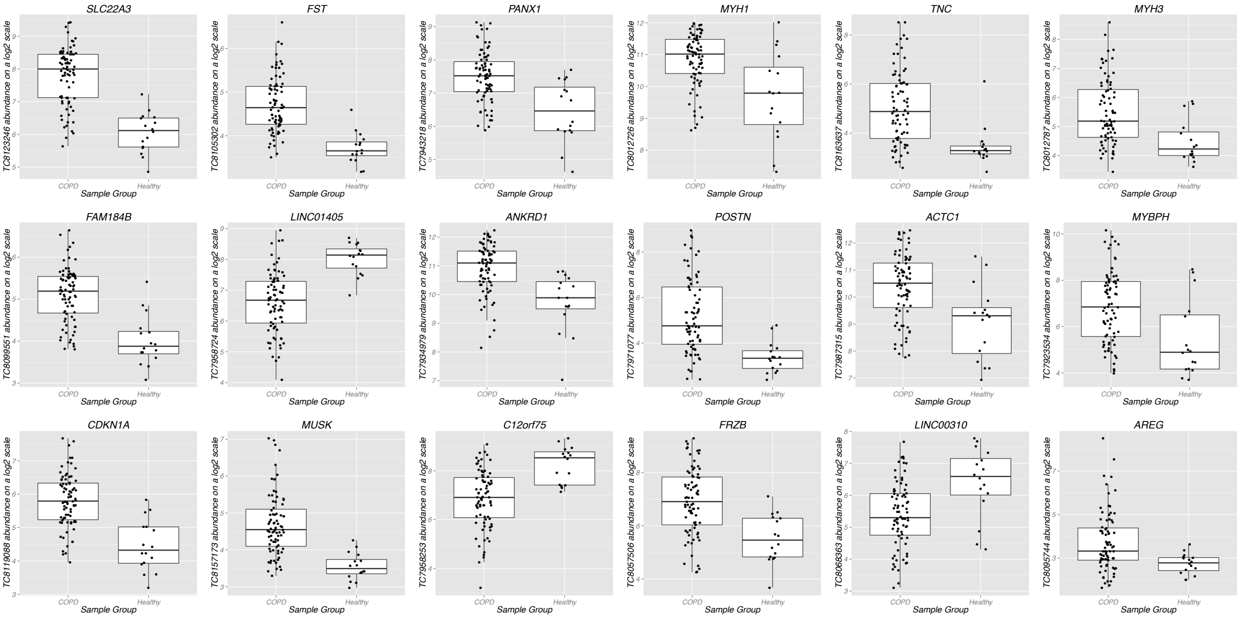

Abbreviations: Transcript Cluster (TC). Abundance is shown on a log2 scale.

Figure E1b: Transcript clusters differentially expressed at a 5% false discovery threshold accompanied by a minimum absolute fold change of 2 split by FFMI in the low and normal range.

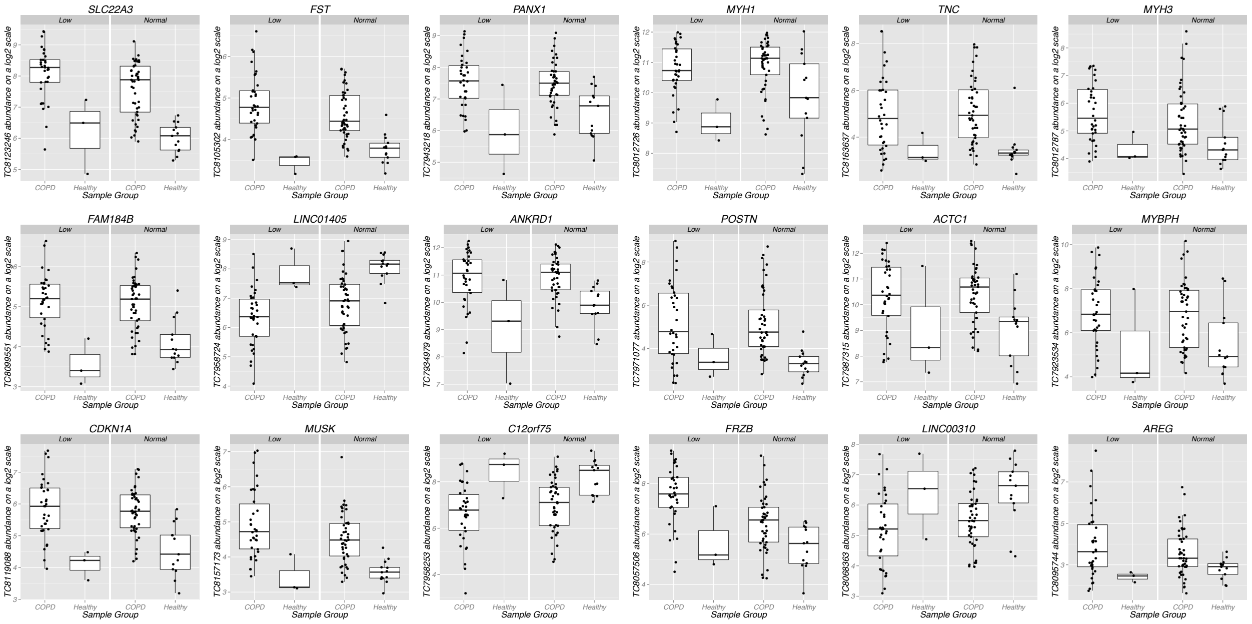

Abbreviations: Transcript Cluster (TC), Fat Free Mass Index (FFMI). Abundance is shown on a log2 scale. A low FFMI and normal FFMI was defined using the Dutch criteria (low if less than 15 kg/m^2 for women and less than 16 kg/m^2 in men).

Figure E2: Full-length IDH2 Western blot image corresponding to Figure 2 A

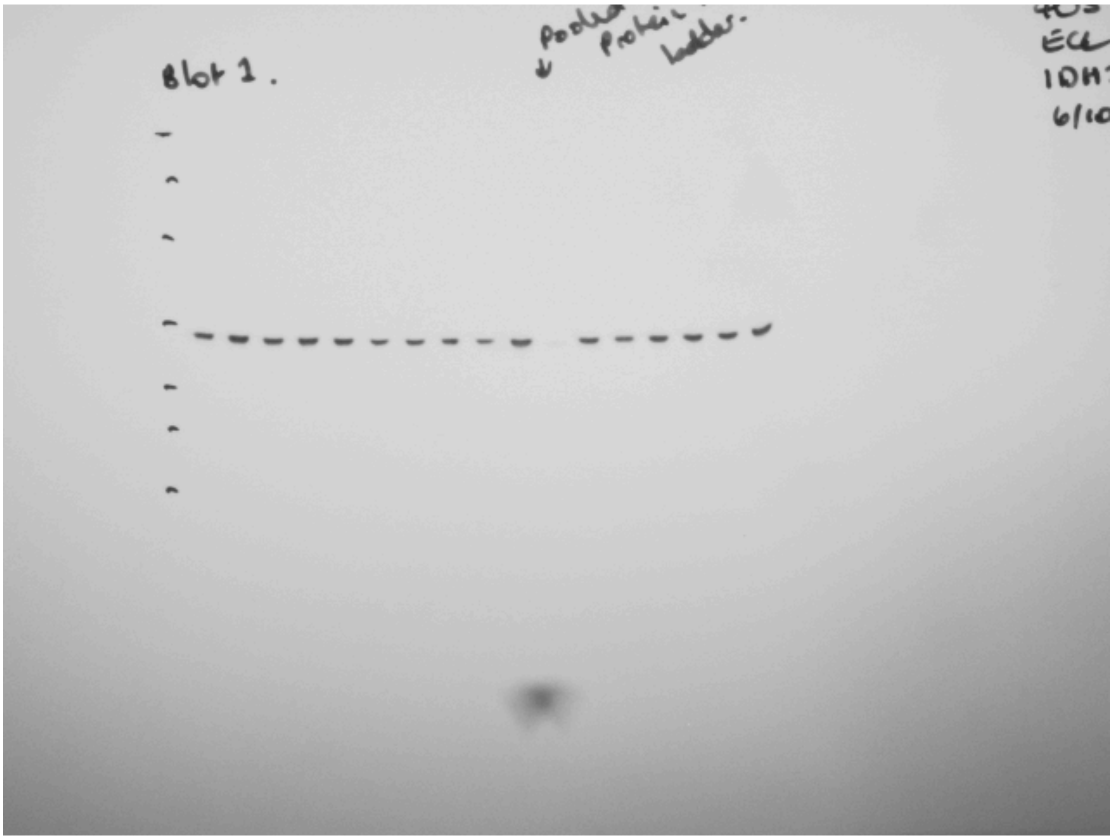

Figure E3: Full-length Ponceau S loading control image corresponding to Figure 2 A

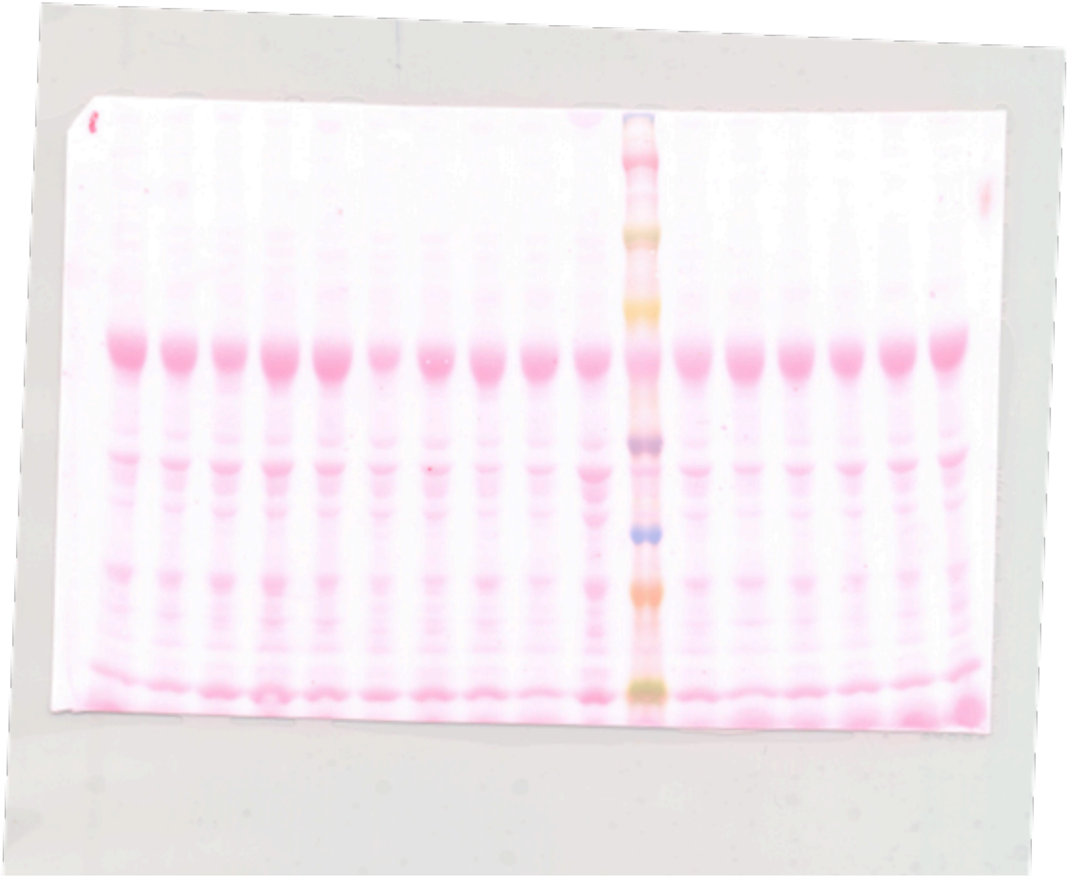

Table E1: The clinical, physiological and quadriceps fibre characteristics of COPD patients and healthy controls.

Group differences in categorical data are tested with Fisher's exact test (†). For quantitative traits, depending on the data distribution values are mean (Standard Deviation), or median (Inter Quartile Range), with the latter denoted by *. Group differences as assessed with the Welch two sample t-test or Mann Whitney U-test (*) depending on the data distribution.

Abbreviations: Forced Expiratory Volume in 1 second (FEV_1), carbon monoxide diffusion capacity (TL_{CO}), arterial partial pressure of oxygen (PaO_2), time for quadriceps force to drop to 80% of initial force in seconds (T_{80}), maximal voluntary contraction (MVC), 6 minute walk test (6MW), oxygen consumption on maximal incremental cycle ergometry (VO_2), cross-sectional area (CSA).

	**COPD patients N=79**	**Healthy controls N=16**	***P*-value**
**Age (years)**	67.08 (8.36)	66.56 (6.86)	7.95E-01
**Sex (% males)†**	64.56	50.00	3.97E-01
**Smoking history (pack-years)***	45.00 (27.25)	3.00 (11.25)	**3.97E-07**
**Current smokers (%)†**	15.79	0.00	1.17E-01
**% on oral prednisolone†**	10.13	0.00	3.44E-01
**FEV_1 (l)***	1.01 (0.69)	2.92 (0.69)	**2.72E-09**
**FEV_1(% predicted)***	42.80 (25.20)	105.50 (10.85)	**3.61E-10**
**TL_{CO}(% predicted)**	42.87 (17.23)	89.69 (16.10)	**3.94E-10**
**PaO_2 (kPa)**	9.23 (1.23)	10.58 (1.30)	**9.62E-04**
**$PaCO_2$ (kPa)**	5.07 (0.51)	5.09 (0.44)	8.95E-01
**Body mass index (kg/m^2)***	24.50 (5.60)	24.80 (3.85)	7.58E-01
**Fat-free mass index (kg/m^2)***	16.00 (2.75)	16.10 (2.30)	4.86E-01
**% with a low fat-free mass index†**	43.04	18.75	9.31E-02
**Quadriceps MVC (kg)***	27.90 (16.70)	33.50 (10.20)	1.31E-01
**Quadriceps twitch force (Kg)***	7.55 (2.40)	8.60 (2.60)	1.98E-01
**Quadriceps endurance (T_{80};s)***	80.00 (27.5)	105.00 (97.50)	1.60E-01
**Locomotion time (min/12 h)***	40.00 (37.50)	90.50 (52.75)	**8.81E-06**
**6MW distance (m)**	392.62 (115.15)	612.31 (70.78)	**1.27E-11**
**Peak VO_2 (ml/kg/min)***	11.40 (5.85)	22.00 (7.73)	**2.43E-08**
**Type I fibre proportion (%)**	28.95 (12.80)	54.44 (15.74)	**7.19E-06**
**Type I/IIa fibre proportion (%)***	3.00 (5.00)	1.50 (4.50)	5.41E-02
**Type IIa fibre proportion (%)**	60.11 (11.67)	40.13 (14.64)	**5.78E-05**
**Type IIx fibre proportion (%)***	4.00 (9.00)	2.50 (4.75)	9.48E-02
**Type I fibre CSA (µm^2)***	5036 (1741)	5120 (1478)	3.32E-01
**Type I/IIa fibre CSA (µm^2)***	5135 (2240)	5487 (2012)	3.44E-01
**Type IIa fibre CSA (µm^2)***	3980 (1748)	4440 (2129)	2.35E-01
**Type IIx fibre CSA (µm^2)***	2909 (1869)	4963 (2953)	**1.42E-03**

Table E2: Demographics of the cohort subset included in the IDH2 protein level analysis

Abbreviations: Forced Expiratory Volume in 1 second (FEV_1), carbon monoxide diffusion capacity (TL_{CO}), arterial partial pressure of oxygen (PaO_2).

	**COPD low N=16**	**COPD normal N=14**	**Healthy controls *N*=13**	***P*-value (between COPD groups)**
**Age (years)**	64(11)	66(10)	69(8)	0.56
**Sex (% males)**	69	43	50	0.27
**Smoking history (pack-years)**	38(28,75)	45(28,71)	4(0,9)	0.89
**Current smokers (%)**	0	14	0	0.21
**FEV_1 (l)**	0.75(0.58,1.3)	1.11(0.93,1.55)	2.92(2.17,3.14)	0.07
**FEV_1(% predicted)**	27(20,46)	47(34,61)	111(101,118)	0.02
**TL_{CO} (% predicted)**	26(18,44)	47(39,56)	92(81,100)	0.007
**Body mass index (kg/m^2)**	22.4(19.7,24.8)	24.3(21.1,26.4)	24.8(22.6,26.5)	0.35
**Fat-free mass index (kg/m^2)**	15.1(14.5,16.4)	15.8(14.1,16.9)	16.4(15.2,19.0)	0.76

Table E3: Enriched Disease Gene Associations (DGA) by Module.
Abbreviations: Unified Medical Language System (UMLS), *P*-value adjusted using the Benjamini and Hochberg method (Adj. *P*), Disease Gene Association (DGA).

ID	Description	*P* value	Adj. *P*	Q value	Count	Module
**umls:C0023264**	Leigh Disease	3.73E-12	1.01E-08	9.28E-09	21	Turquoise
**umls:C0878544**	Cardiomyopathies	5.88E-09	7.99E-06	7.31E-06	46	Turquoise
**umls:C2936907**	NADH:Q(1) Oxidoreductase deficiency	1.79E-08	1.63E-05	1.49E-05	9	Turquoise
**umls:C0949658**	Cardiomyopathy, Hypertrophic, Familial	4.92E-08	3.34E-05	3.06E-05	13	Turquoise
**umls:C0342776**	Nicotinamide adenine dinucleotide coenzyme Q reductase deficiency	2.43E-07	1.32E-04	1.21E-04	11	Turquoise
**umls:C0751651**	Mitochondrial Diseases	2.99E-07	1.36E-04	1.24E-04	20	Turquoise
**umls:C0011853**	Diabetes Mellitus, Experimental	5.07E-07	1.97E-04	1.80E-04	42	Turquoise
**umls:C1838979**	MITOCHONDRIAL COMPLEX I DEFICIENCY	1.09E-06	3.70E-04	3.38E-04	9	Turquoise
**umls:C0007193**	Cardiomyopathy, Dilated	1.58E-06	4.77E-04	4.36E-04	36	Turquoise
**umls:C0017924**	Glycogen Storage Disease Type V	1.69E-05	4.61E-03	4.21E-03	6	Turquoise
**umls:C0007194**	Hypertrophic Cardiomyopathy	3.01E-05	7.45E-03	6.82E-03	20	Turquoise
**umls:C0034345**	Pyruvate Dehydrogenase Complex Deficiency Disease	5.20E-05	1.18E-02	1.08E-02	6	Turquoise
**umls:C0026848**	Myopathy	7.40E-05	1.53E-02	1.40E-02	26	Turquoise
**umls:C0162670**	Mitochondrial Myopathies	7.89E-05	1.53E-02	1.40E-02	10	Turquoise
**umls:C1960469**	Left ventricular non compaction	9.75E-05	1.68E-02	1.53E-02	6	Turquoise
**umls:C0022548**	Keloid	9.85E-05	1.68E-02	1.53E-02	18	Turquoise
**umls:C0003811**	Cardiac Arrhythmia	1.21E-04	1.93E-02	1.77E-02	10	Turquoise
**umls:C0340489**	Lone atrial fibrillation	1.70E-04	2.50E-02	2.29E-02	5	Turquoise
**umls:C1271104**	Blood pressure finding	1.75E-04	2.50E-02	2.29E-02	30	Turquoise
**umls:C0940937**	Precancerous lesions	1.88E-04	2.56E-02	2.34E-02	13	Turquoise
**umls:C0342782**	Depletion of mitochondrial DNA	2.20E-04	2.82E-02	2.58E-02	6	Turquoise
**umls:C0242656**	Disease Progression	2.28E-04	2.82E-02	2.58E-02	21	Turquoise
**umls:C0085548**	Autosomal Recessive Polycystic Kidney Disease	2.57E-04	3.04E-02	2.78E-02	8	Turquoise
**umls:C0949857**	Mitochondrial Respiratory Chain Deficiencies	2.80E-04	3.17E-02	2.90E-02	6	Turquoise
**umls:C0268237**	Cytochrome-c Oxidase Deficiency	3.01E-04	3.28E-02	3.00E-02	8	Turquoise
**umls:C0029124**	Optic Atrophy	3.52E-04	3.68E-02	3.37E-02	7	Turquoise
**umls:C0018800**	Cardiomegaly	3.90E-04	3.93E-02	3.59E-02	18	Turquoise
**umls:C0020295**	Hydronephrosis	4.38E-04	4.26E-02	3.89E-02	6	Turquoise
**umls:C1511291**	Breast Cancer Model	4.67E-04	4.36E-02	3.98E-02	10	Turquoise
**umls:C0020615**	Hypoglycemia	4.80E-04	4.36E-02	3.98E-02	15	Turquoise
**umls:C0085584**	Encephalopathies	5.36E-04	4.53E-02	4.14E-02	14	Turquoise
**umls:C0162292**	External Ophthalmoplegia	5.49E-04	4.53E-02	4.14E-02	4	Turquoise
**umls:C0221155**	Systolic hypertension	5.49E-04	4.53E-02	4.14E-02	4	Turquoise
**umls:C0034069**	Pulmonary Fibrosis	1.51E-08	1.82E-05	1.49E-05	13	Green
**umls:C0024667**	Animal Mammary Neoplasms	5.16E-07	3.11E-04	2.54E-04	9	Green
**umls:C0042373**	Vascular Diseases	2.98E-06	1.20E-03	9.78E-04	11	Green
**umls:C1333768**	Gastric Gastrointestinal Stromal Tumor	1.75E-05	5.08E-03	4.15E-03	3	Green
**umls:C0030524**	Paratuberculosis	2.11E-05	5.08E-03	4.15E-03	4	Green
**umls:C0239946**	Fibrosis, Liver	2.82E-05	5.58E-03	4.56E-03	9	Green
**umls:C1800706**	Idiopathic Pulmonary Fibrosis	3.65E-05	5.58E-03	4.56E-03	9	Green
**umls:C0025286**	Meningioma	4.09E-05	5.58E-03	4.56E-03	10	Green

**umls:C0278996**	Cancer of Head and Neck	4.17E-05	5.58E-03	4.56E-03	10	Green
**umls:C0024115**	Lung diseases	5.58E-05	6.07E-03	4.96E-03	9	Green
**umls:C0024668**	Mammary Neoplasms, Experimental	6.40E-05	6.07E-03	4.96E-03	7	Green
**umls:C0032927**	Precancerous Conditions	6.69E-05	6.07E-03	4.96E-03	10	Green
**umls:C0026640**	Mouth Neoplasms	7.01E-05	6.07E-03	4.96E-03	7	Green
**umls:C1335110**	Oligodendroglial Neoplasm	7.05E-05	6.07E-03	4.96E-03	3	Green
**umls:C1334015**	High Grade Intraepithelial Neoplasia	1.17E-04	9.40E-03	7.68E-03	3	Green
**umls:C0553692**	Brain hemorrhage	2.05E-04	1.54E-02	1.26E-02	3	Green
**umls:C0162871**	Aortic Aneurysm, Abdominal	2.82E-04	2.00E-02	1.63E-02	6	Green
**umls:C0206638**	Giant Cell Tumor of Bone	3.27E-04	2.09E-02	1.71E-02	4	Green
**umls:C0494165**	Secondary malignant neoplasm of liver	3.29E-04	2.09E-02	1.71E-02	9	Green
**umls:C1292772**	Leukemia, Myeloid, Chronic, Atypical, BCR-ABL Negative	3.63E-04	2.15E-02	1.76E-02	3	Green
**umls:C0023269**	Leiomyosarcoma	3.75E-04	2.15E-02	1.76E-02	5	Green
**umls:C0877008**	Enzyme inhibition disorder	5.35E-04	2.93E-02	2.40E-02	3	Green
**umls:C0022572**	Keratoacanthoma	5.85E-04	3.06E-02	2.51E-02	3	Green
**umls:C0282612**	Prostatic Intraepithelial Neoplasias	6.24E-04	3.13E-02	2.56E-02	5	Green
**umls:C0032285**	Pneumonia	7.30E-04	3.52E-02	2.88E-02	8	Green
**umls:C0010072**	Coronary Thrombosis	8.78E-04	3.94E-02	3.22E-02	3	Green
**umls:C0007786**	Brain Ischemia	8.83E-04	3.94E-02	3.22E-02	8	Green
**umls:C0699893**	Skin carcinoma	1.07E-03	4.60E-02	3.76E-02	4	Green
**umls:C1834674**	Bethlem myopathy	1.23E-03	4.90E-02	4.01E-02	2	Green
**umls:C0029456**	Osteoporosis	1.27E-03	4.90E-02	4.01E-02	8	Green
**umls:C0993582**	Arthritis, Experimental	1.29E-03	4.90E-02	4.01E-02	5	Green
**umls:C0000786**	Spontaneous abortion	1.30E-03	4.90E-02	4.01E-02	6	Green
**umls:C1519670**	Tumor Angiogenesis	1.34E-03	4.90E-02	4.01E-02	8	Green
**umls:C0349782**	Ischemic cardiomyopathy	1.58E-07	3.29E-04	2.86E-04	9	Blue
**umls:C0221505**	Lesion of brain	1.47E-05	1.34E-02	1.16E-02	7	Blue
**umls:C0600452**	Hepatopulmonary Syndrome	1.94E-05	1.34E-02	1.16E-02	8	Blue
**umls:C0026848**	Myopathy	4.53E-05	2.35E-02	2.04E-02	13	Blue
**umls:C0686353**	Muscular Dystrophies, Limb-Girdle	1.13E-04	4.61E-02	4.00E-02	6	Blue
**umls:C0006287**	Bronchopulmonary Dysplasia	1.35E-04	4.61E-02	4.00E-02	10	Blue
**umls:C0042345**	Varicosity	1.65E-04	4.61E-02	4.00E-02	5	Blue
**umls:C0026846**	Muscular Atrophy	2.22E-04	4.61E-02	4.00E-02	9	Blue
**umls:C1511789**	Desmoplastic	2.30E-04	4.61E-02	4.00E-02	6	Blue
**umls:C0007193**	Cardiomyopathy, Dilated	2.40E-04	4.61E-02	4.00E-02	14	Blue
**umls:C0011884**	Diabetic Retinopathy	2.77E-04	4.61E-02	4.00E-02	10	Blue
**umls:C0026850**	Muscular Dystrophy	2.98E-04	4.61E-02	4.00E-02	10	Blue
**umls:C0410179**	Scleroatonic muscular dystrophy	3.10E-04	4.61E-02	4.00E-02	3	Blue
**umls:C0877009**	Muscle fibrosis	3.10E-04	4.61E-02	4.00E-02	3	Blue
**umls:C0149721**	Left Ventricular Hypertrophy	3.42E-04	4.74E-02	4.12E-02	10	Blue
